# The Terrific Skink bite force suggests insularity as a likely driver to exceptional resource use

**DOI:** 10.1038/s41598-022-08148-6

**Published:** 2022-03-17

**Authors:** Michael J. Jowers, Yuri Simone, Anthony Herrel, M. Pilar Cabezas, Raquel Xavier, Magaly Holden, Renaud Boistel, John C. Murphy, Mathieu Santin, Stephane Caut, Renoir J. Auguste, Arie van der Meijden, Franco Andreone, Ivan Ineich

**Affiliations:** 1 CIBIO, Centro de Investigação em Biodiversidade e Recursos Genéticos, InBIO Laboratório Associado, Campus de Vairão, Universidade do Porto, 4485-661 Vairão, Portugal; 2 BIOPOLIS Program in Genomics, Biodiversity and Land Planning, CIBIO, Campus de Vairão, 4485-661 Vairão, Portugal; 3 Département Adaptations du Vivant, UMR 7179 CNRS/MNHN, 57 Rue Cuvier, Case postale 55, 75231 Paris Cedex 5, France; 4 Evolutionary Morphology of Vertebrates, Ghent University, Campus Ledeganck, K.L. Ledeganckstraat 35, 9000 Gent, Belgium; 5 Faculdade de Ciências da Universidade do Porto, Rua do Campo Alegre s/n, 4169-007 Porto, Portugal; 6 Centre d’Écologie Fonctionnelle et Évolutive (UMR CNRS 5175), École Pratique des Hautes Études, Biogéographie et Écologie des Vertébrés, Campus CNRS, Montpellier, France; 7 Science and Education, Field Museum, 1400 Lake Shore Dr., Chicago, IL 60605 USA; 8 Inserm U 1127, CNRS UMR 7225, Centre for NeuroImaging Research, ICM (Brain and Spine Institute), Sorbonne University, Paris, France; 9 Institut du Cerveau – Paris Brain Institute – ICM, INSERM, CNRS, Sorbonne Université, 75013 Paris, France; 10 ANIMAVEG Conservation, 58 Avenue Allende, 94800 Villejuif, France; 11 Department of Life Science, The University of the West Indies, St. Augustine, Trinidad and Tobago; 12 Museo Regionale di Scienze Naturali, Via G. Giolitti, 36, 10123 Turin, Italy; 13 Institut de Systématique, Évolution, Biodiversité (ISYEB), Muséum National d’Histoire Naturelle, Sorbonne Université, École Pratique des Hautes Études, CNRS, Université des Antilles, CP 30, 57 Rue Cuvier, 75005 Paris, France

**Keywords:** Biodiversity, Ecology, Evolution, Physiology, Zoology

## Abstract

Natural history museum collections hold extremely rare, extinct species often described from a single known specimen. On occasions, rediscoveries open new opportunities to understand selective forces acting on phenotypic traits. Recent rediscovery of few individuals of Bocourt´s Terrific Skink *Phoboscincus bocourti*, from a small and remote islet in New Caledonia allowed to genetically identify a species of land crab in its diet. To explore this further, we CT- and MRI-scanned the head of the holotype, the only preserved specimen dated to about 1870, segmented the adductor muscles of the jaw and bones, and estimated bite force through biomechanical models. These data were compared with those gathered for 332 specimens belonging to 44 other skink species. Thereafter we recorded the maximum force needed to generate mechanical failure of the exoskeleton of a crab specimen. The bite force is greater than the prey hardness*,* suggesting that predation on hard-shelled crabs may be an important driver of performance. The high bite force seems crucial to overcome low or seasonal variations in resource availability in these extreme insular environments. *Phoboscincus bocourti* appears to be an apex predator in a remote and harsh environment and the only skink known to predate on hard-shelled land crabs.

## Introduction

The International Union for the Conservation of Nature (IUCN) declared 160 animal and plant species extinct in the last decade (2010–2019), but estimates suggest this may be as high as one thousand species per year^1–3^. The biological information that could have been gathered from now extinct species is irreplaceable and lost forever. Such species are often so rare that their only confirmed occurrence is from their initial description. Nevertheless, some species declared extinct are occasionally rediscovered many years later, and sometimes called Lazarus taxa. This happens because these species mostly occur in the understudied biomes^4,5^ and many species descriptions are based solely on the holotype. Their restricted ranges and remote localities make biodiversity surveys challenging and remain the main reason for our lack of knowledge. On average, rediscoveries are made about 60 years after the Lazarus taxon has been described^6^.

**Figure 1:**
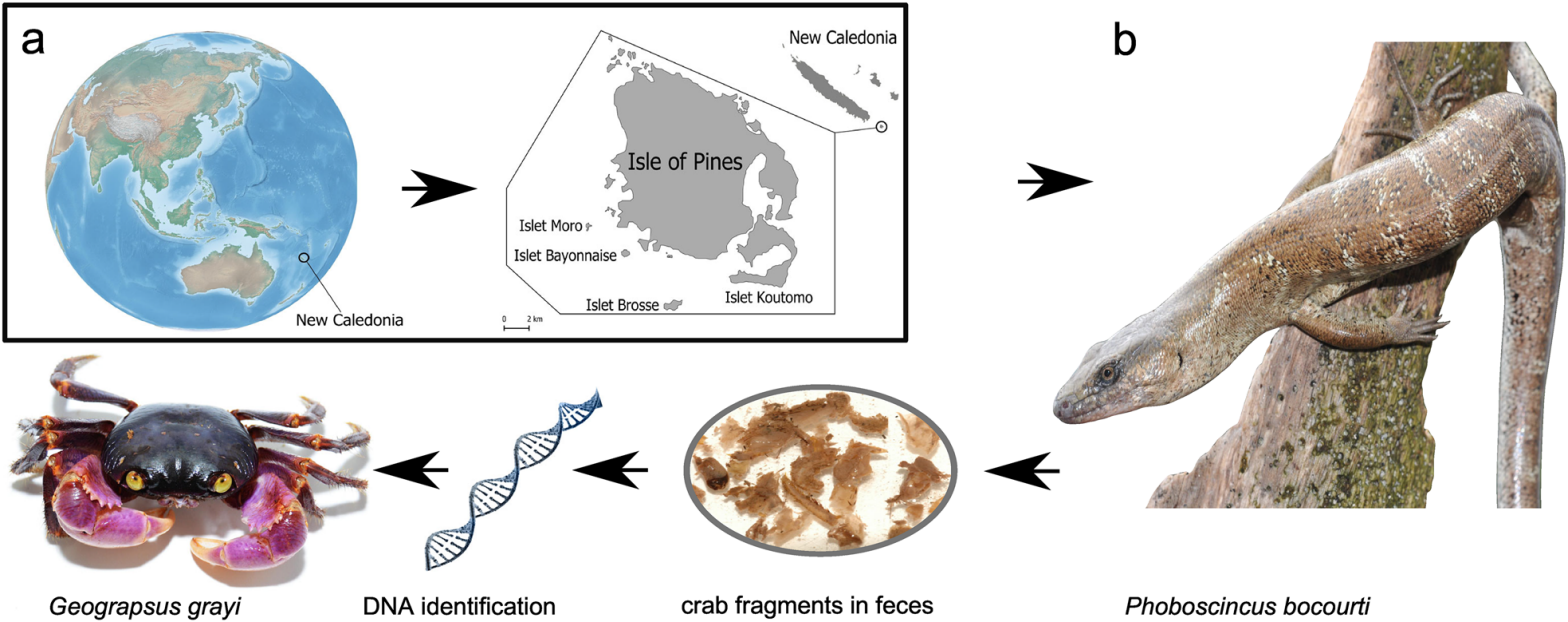
(**a**) Map of New Caledonia, Isle of Pines and satellite islands including the islet where *Phoboscincus bocourti* is found. The map was generated using QGIS v3.10 software (https://www.qgis.org/en/site/), with the Cross-blended hypsometric tint Earth layer downloaded from Natural Earth (https://www.naturalearthdata.com/). The New Caledonia and Isle of Pine layers were generated using QGIS built in features. (**b**) Picture of *P. bocourti,* and crab remains found in its feces and genetic confirmation of crab identification as *Geograpsus grayi*. Picture credits; (*P. bocourti*—Ivan Ineich, and *G. grayi*—Martin Höhle).

**Figure 2:**
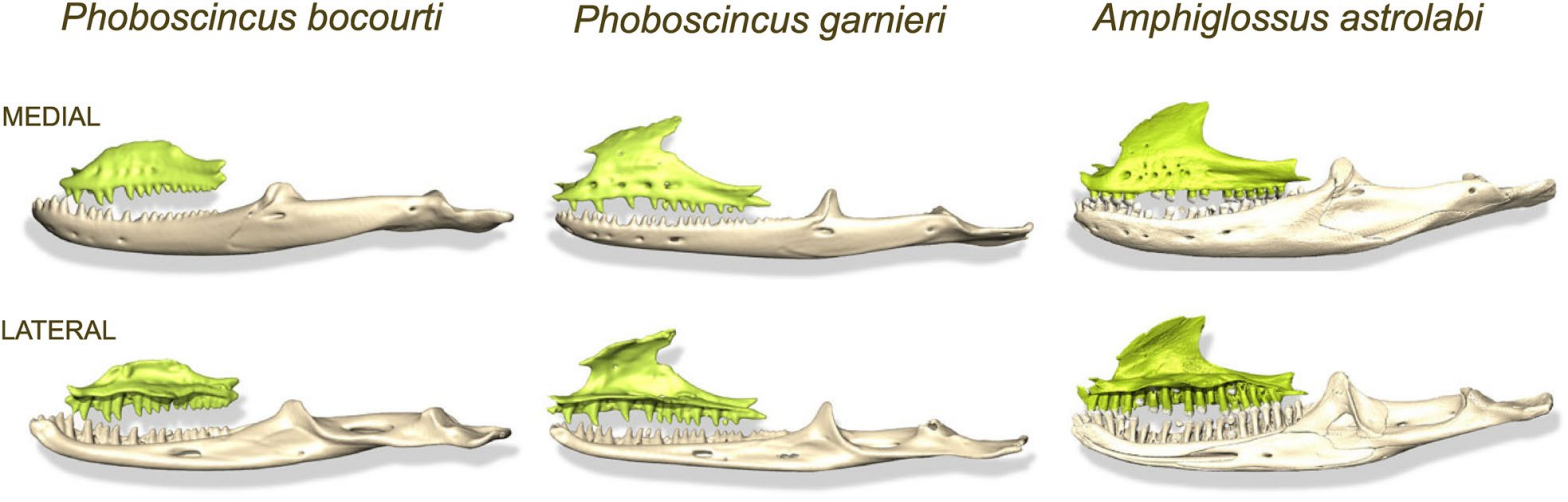
Figure showing different top mandible (in green) and bottom mandible (in white) for *Phoboscincus bocourti, P. garnieri* and *Amphiglossus astrolabi* (Beamline ID19).

An extraordinary example of a rediscovered species is Bocourt´s Terrific Skink *Phoboscincus bocourti* (Brocchi, 1876). It was only known from its holotype (supplementary Fig. S1), captured around 1870 in New Caledonia with no further precise locality data, but most likely captured on the largest island called Grande Terre^7^. In 2000, a juvenile was found dead, but not collected, on the beach on an islet off Île des Pins. It was likely prey dropped by a raptor. The first live and immediately correctly identified specimen was found in 2003 by one of us (II) and released. Subsequently one individual was captured and released in 2005, (II), seven other specimens (three captured and released and four more were photographed) in 2012 (by, MH and II), and another individual, illegally collected, in 2018^8^. Of the nearly 100 lizards endemic to New Caledonia and the 19 species native to the Île des Pins (Isle of Pins) (152 km^2^)^9^, only *P. bocourti* is restricted to one satellite islet of the Isle of Pins (less than 1 km^2^ area). Although this species is most likely present in several other surrounding islets^10^, it is one of the most geographically restricted endemic skink species in the world (Fig. 1). The unusual large size of the holotype [50 cm total length, 28 cm snout-vent length (SVL)] and curved anterior teeth gave rise to the generic common name, the Terrific Skink (AKA the Terror Skink). Intriguingly, such predatory-type dentition is often associated with the smaller insectivorous skinks, as teeth in larger species tend to have a cylindrical shaft with rather blunt conical tips for a diet that is more plant based. The more widespread Giant Skink, *Phoboscincus garnieri* (Bavay, 1869) (~ 20 cm SVL), the only other species within the genus^11^, has a very different dentition (Fig. 2), with rather blunt teeth compared to *P. bocourti.* This dentition structure suggests a different feeding ecology. Clues to the diet of *P. bocourti*, and hence the function of its unusual teeth can be derived from recent isotope analyses on the 2005 and 2012 individuals as well as the holotype, which suggested it fed on smaller skinks^12^.

*Phoboscincus bocourti*´s unusual dental morphology might have been shaped by island isolation, habitat use, and trophic niche (changes in food availability possibly due to seasonal variations). Insularity affects many aspects of the biology of lizards such as body size, antipredator behavior and morphology^13–16^. Moreover, lizard feeding ecology on islands is significantly different from that on the mainland for a same species^17–21^. Different prey communities, their abundance and seasonal variation on islands impose changes in a lizard’s dietary niche and often lead to the utilization of novel resources^22^, a powerful driver of morphological evolution. Head size (width and height) is known to be associated with bite force, which in turn is related to foraging, mating, and fighting^13,23^. Discerning between these factors is difficult but essential to disentangle the evolutionary processes driving morphological variation in different ecological contexts^24^. The large body size and relatively large head size, combined with its sharp dentition, make *P. bocourti* a likely apex predator. Furthermore, *P. bocourti* is found on a small and remote oceanic island, two factors known to significantly impact the diversity and abundance of prey and vegetation^25^ and thus conditioning resource availability and population density^26^. This may lead to a greater competition for resources^27^ and possibly, it may drive the evolution of bite force^22^ and body size^28^. Both features are essential to deal with new/different prey types. The association between the ability to deal with armored prey and increase in bite force has been documented for lacertids, as well as other vertebrates^29–36^, suggesting that *P. bocourti* may have evolved increased bite force in response to low or season-dependent availability of resources and the exploitation of new prey types to survive.

New observations from the rediscovered population offer a unique opportunity to address long-standing questions regarding *Phoboscincus bocourti*´s ecology and evolution. Here we used molecular analyses to genetically identify prey item remains in the only two fecal samples retrieved from two individuals caught in the field in 2012 by two of us (MH and II). We conducted MRI analyses on the holotype, allowing the reconstruction of the soft tissues and the modelling of bite force in this enigmatic species. Moreover, bite force estimates for *P. bocourti* were compared to data obtained in vivo for other species of skinks, including the largest within the group ^37^.

## Results

### Fecal sample DNA identification

Universal primers failed to amplify any crustacean samples and therefore specific primers were employed (Supplementary Table S1). Both samples amplified the same haplotypes for both mitochondrial markers. GenBank Blast searches matched the land crab *Geograpsus grayi*^38^. The COI (264 base pair—bp) fragment matched 100% while the 16S rDNA (251 bp) fragment matched 98% to *G. grayi.* Adult *G. grayi* can reach a size of 6 cm across the carapace^39^, which indicates that the specimen used for analyses was likely an adult (4.5 cm, Fig. 1).

### CT and MRI scanning and segmentation

**Figure 3:**
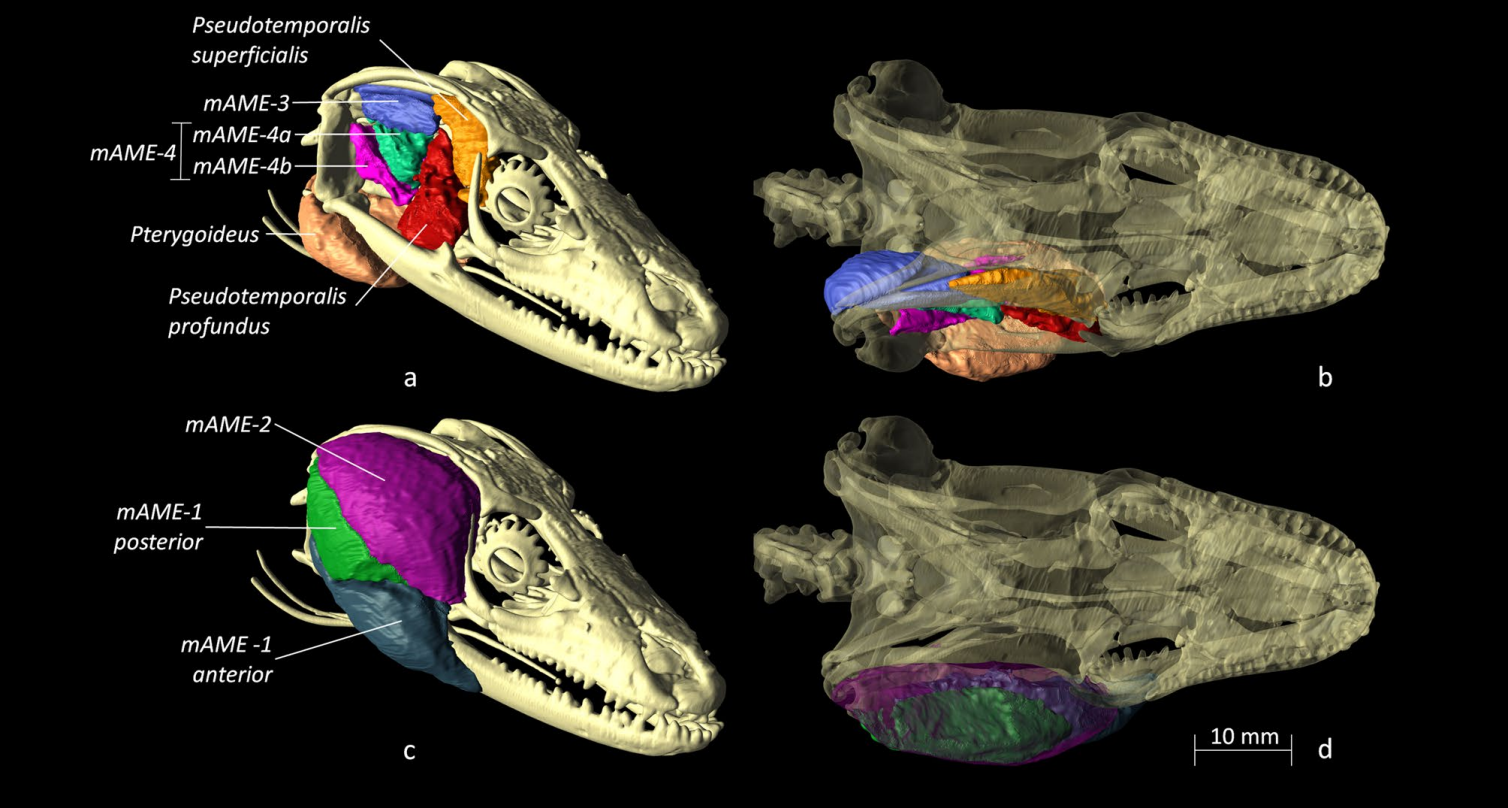
Reconstruction of the three major groups of jaw adductors from CT and MRI scanning and segmentation of the holotype of *Phoboscincus bocourti.* (**a**,**b**) Lateral and dorsal views of the most internal sets of jaw adductors: *pseudotemporalis* complex composed by the muscles *pseudotemporalis profundus* in red, *pseudotemporalis superficialis* in orange. The m. *pterygoideus* is colored in salmon. The medial portion of the mAME *medialis* (mAME-3) is shown in blue. The mAME *profundus* (mAME-4) complex is also composed by two bundles respectively colored in aquamarine green (mAME-4a) and fuchsia (mAME-4b). (**c**,**d**) lateral and dorsal views of the mAME *superficialis* (mAME-1) and the radial portion of the mAME *medialis* (mAME-2) complexes. The mAME-1 is composed by the anterior bundle (dark blue) and the posterior bundle (dark green). The mAME-2 is instead shown colored in violet. In the lateral views the transparency of the skull has been increased to enhance the visibility of the position of the muscles.

From the segmentation of the MRI, the three major groups of jaw adductors were reconstructed (Fig. 3). The jaw muscles are similar to previous descriptions of the jaw adductors in scincid lizards^40,41^. The jaw musculature (Fig. 3) is well developed and consists of external (m. *adductor mandibulae externus,* mAME), internal (pseudotemporalis and pterygoideus muscles) muscle groups clearly visible in the segmentation. The external adductor consists of a superficial, a medial, and a profundus part which together represent more than the half (56%) of the total volume of all the adductor muscles, slightly larger than in two other scincids of similar size (*T. scincoides*: 52.9%; *C. zebrata*: 55.6%; see ^42^). The superficial part corresponds to the m. adductor mandibulae externus 1, the medial part to the m. adductor mandibulae externus 2 and 3, and the profundus part to the m. adductor mandibulae externus 4. The internal adductor in *P. bocourti* is comparable to the same muscle groups as in other scincids and consists of the pseudotemporal (superficial and profundus parts) and the pterygoid muscles (lateral and medial parts). The pterygoid muscle accounts for 35% of the volume of the jaw adductors similar to *T. scincoides* (34.6%) but slightly larger than what is observed for *C. zebrata* (28.5% see^42^). The posterior adductor was impossible to separate based on the MRI data in *P. bocourti.* In most scincid lizards this muscle originates at the anterolateral side of the quadrate and is covered by the quadrate aponeurosis at its dorsal side. The fibres then run anteroventrally and insert on the dorsal side of the lower jaw, behind the coronoid bone. In our segmentation this part was included as the deepest fibers of the MAME1 anterior.

### *Phoboscincus bocourti* bite force and prey hardness

**Figure 4:**
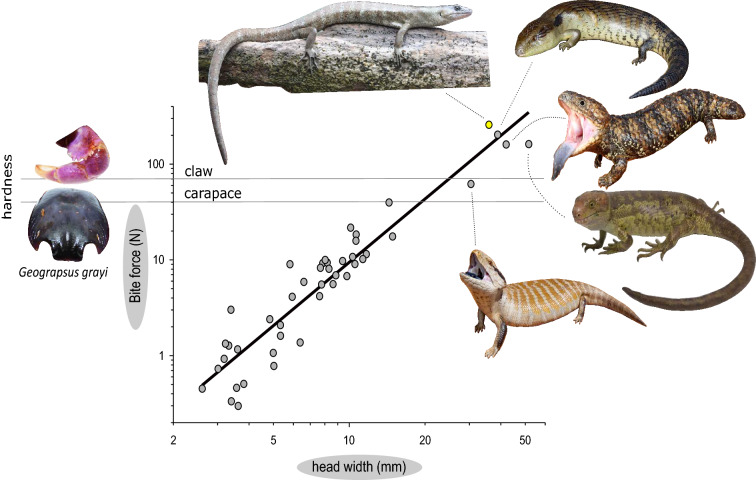
Graph showing head width versus bite force to compare the bite force data estimated for *Phoboscincus bocourti* and 332 specimens across 44 skink species at the tip of the jaw and at the gape angle of 30°. The skinks depicted on the graph are: *P. bocourti* on top (photo credit Ivan Ineich), *Tiliqua scincoides* second right, *T. rugosa* third bottom, *Corucia zebrata* fourth bottom (photo credit David Utrera), *T. multifasciata* fifth bottom (Photo credit for *Tiliqua *spp. Anders Zimny).

Bite forces calculated at the back of the tooth row at ten degrees of jaw opening amounted up to 256 N (newtons) and reduced to 143 N (56% of the max. bite force) at the front of the tooth row, 178 N (69% of the max. biteforce) at the posterior caniniform tooth and finally 172 N (67% of the max. biteforce) at the anterior caniniform tooth. When opening the jaws biteforces at the back of the tooth row decreased to 229 N (89% of max. force) at a gape angle of 30°, and to 159 N (62% of max. force) for a gape angle of 60°. At the front of the jaw bite force reduced to 128 N (50% of max. force) at 30° and 89 N (34% of max. force) at 60° of jaw opening. At the posterior caniniform tooth biteforce is 159 N (62% of the max. bite force) for a gape angle of 30° and 110 N (43% of the max. biteforce) for a gape angle of 60°. At the anterior caniniform tooth biteforce is 154 N (60% of the max. bite force) for a gape angle of 30° and 107 N (41% of the max. biteforce) for a gape angle of 60°. Whereas *P. bocourti* thus maintains bite force relatively well at medium gape angles it suffers from a significant decrease in force at larger gapes. When plotting the calculated bite force against the in vivo measured forces for other species of skink (Fig. 4) *Phoboscincus bocourti* is clearly situated above the regression line when using bite forces calculated at back of the tooth row. Yet, when using the forces calculated at the front of the tooth row (i.e., where in vivo bite forces are measured for other species) it falls perfectly on the regression line suggesting an average bite force for its head width. It is worth mentioning that when superimposing the hardness of fresh non-molting crab claws upon the bite force graph this falls within the reach of the bite force of *P. bocourti* (see Fig. 4). Note, however, that the crab tested was 4.5 cm, while they can reach up to 6 cm^39,43^ and that hardness increases linearly with size. Consequently, the hardness of large adult crabs perfectly matches the bite forces *P. bocourti* is capable of generating. Note that the shape of the teeth is different from the shape of our hardness testing device and the pointed teeth will concentrate the force on a smaller area. Our estimate of the bite force required to break a crab claw by *P. bocourti* is therefore likely an overestimate; it may be able to break crab claws at a lower force.

## Discussion

Digestive track and fecal analyses are traditionally used to determine diet of animals coming from museum collections, but when specimens are in short supply these techniques may result in documenting a rare or uncommon meal. The use of stable isotopes provides time-integrated information on assimilated foods over a period of time. However, small variations in isotopic discrimination factors (i.e., isotope ratios) can lead to large errors or meaningless results from isotopic models^44,45^. Stable isotope processes are not well described in reptiles, which limits reliable inferences on trophic and nutrient dynamics. To date, only a few studies have estimated these parameters in terrestrial reptiles^46–50^ making analyses difficult. For example, Steinitz et al.^47^ found significant differences in isotopes among tissues and between juvenile and adult *Iguana*. Similarly, Lattanzio and Miles^48^ found very different estimated discrimination factors in a small-bodied, insectivorous lizard (*Urosaurus ornatus*). In the only study on the diet of *Phoboscincus bocourti*, the estimates of the isotope discriminating factors were not available^12^. The correction estimates suggested a diet on skinks, likely a misleading result due to a correction applied or to a seasonal variation in crab feeding^12^. Moreover, under the assumption of a low isotope enrichment^48^, *P. bocourti* seems to be a predator of terrestrial crabs (*Geograpsus grayi*) and of the giant New Caledonian endemic diplodactylid gecko *Rhacodactylus leachianus* (Cuvier, 1829) (Supplementary Fig. S2). It is worth mentioning that in their isotope analyses, Caut et al.^12^ showed that *P. bocourti* does not feed on plant material, which is often associated with higher bite forces, especially in lizards^13,23,31,36,51–54^. It is also worth mentioning that *Phoboscincus bocourti* fecal pellets only contained parts of a crab and therefore it is not possible to ascertain if each pellet was the only deposition of the last meal or if it was the whole meal. Chitin is more resistant to digestion than other biological tissues and thus it persists longer in the stomach and can be at the origin of an observation artifact due to other softer tissues being digested first.

The enigmatic Terrific Skink earned its name because of its predatory appearance, large size, and unusual sharp caniniform teeth according to the original genus description^55^. However, until its rediscovery in 2003, there was no natural history information on the species, and it remained a mysterious, poorly known, believed to be extinct reptile even described as nocturnal (see review in^7^). This study highlights the importance of combining historical museum collections with contemporary surveys to piece together the natural history of rare species. Our data suggest that *P. bocourti* has a high bite force relative to its size, on top of being among the larger species of skink^37^. The bite force calculated appears to be ecologically relevant, as it may allow access to an underutilized resource (Fig. 4). The presence of two lateral canines (Supplementary Fig. S3) seems unique in *P. bocourti*, which might relate to its diet, although a possible role in sexual selection cannot be discarded.

Few reptiles feed on crabs, and these are often large species with extreme bite force such as crocodiles, turtles, and tortoises^56–58^ but several snakes consume crabs as part of their diet. Some of these snakes are crustacean specialists. Homalopsid snakes of the genera *Fordonia* and *Gerarda* are known for eating crabs^59,60^. *Fordonia leucobalia* consumes hard-shelled crabs, and has unusually robust rear-fangs and a thickened stomach lining that are considered adaptations for this diet^40,57–64^. *Gerarda prevostiana* eats only crabs in the process of molting their exoskeleton. Other homalopsid snakes are reported to eat crabs on occasion (e.g. *Enhydris bennetti*; see Lau & Melville^65^). Some dipsadid snakes such as *Tretanorhinus variabilis* are also known to feed on crustaceans^66^, as do some natricid snakes of the genera *Reginae* and *Liodytes*^67^ both of which are crustacean specialists. *Reginae* feeds on soft-shelled crustaceans while *Liodytes* preys on hard-shelled crustaceans. Lizards reported to eat crabs include members of the families Varanidae, Iguanidae, Leiocephalidae, Tropiduridae, Scincidae, Teiidae, Dactyloidae and Anguidae (Supplementary Table S2).

Despite more than 1700 species of skinks, there are surprisingly few records of skinks eating crabs. This scarcity might be due to the fact that relatively few skinks are likely to encounter crabs unless they are aquatic or inhabit shorelines, mangroves or forests where terrestrial and semi-terrestrial crabs are abundant. The scarcity of records may also be related to the lack of diet information for most of the species, particularly for large coastal species and aquatic species more generally. Most importantly, skinks need to be large enough to deal with larger crabs. Data on skinks feeding on crabs are available for the semi-aquatic Diving Skink, *Amphiglossus astrolabi*, endemic to Madagascar^68^, that feeds on mangrove crabs (Fig. 2). Computerized tomography (CT scan) of *Amphiglossus reticulatus* has similarly revealed crab remains in the stomach (Supplementary Fig. S4). The Mangrove Skink, *Emoia atrocostata*, a medium sized species, has been reported feeding on small land crabs (< 2 cm)^69–71^ as does the Reclusive Litter Skink, *Eugongylus albofasciolatus* (Supplementary Fig. S5) (see^70^) from the Solomon Islands. All Anguidae, Iguanidae, and Scincidae that are known to feed on crabs are found on islands, mostly on small isolated islands (Supplementary Table S2). Some teiids found in sympatry with crabs in flooded forests, mangroves, and marshes, have incorporated crabs into their diet. Notably, to the best of our knowledge the only reports of scincids feeding on land crabs are from small remote tropical islands such as the Solomon Islands and the small islet in New Caledonia where the Terrific Skink occurs.

The Oceanic Land Crab, *Geograpsus grayi* (Edwards, 1853) is the most terrestrial member of the genus. It can weigh up to 50 g, it uses tree holes and rock crevices, climbs well, and is nocturnal^38^. It is endemic to the Indian and Pacific Oceans. Females move in mass to the ocean to spawn in December^38,72,73^. While *Geograpsus grayi* is prey for *Phoboscincus bocourti* it is unclear whether crabs are eaten during molting or not (or both), or if predation remains opportunistic to prey availability and to possibly seasonal availability. *Phoboscincus bocourti*´s isotope analyses revealed a diet of *Geograpsus grayi* but also the arboreal and mostly nocturnal New Caledonian Giant Gecko, *Rhacodactylus leachianus*^12^, a large species (360 mm total length) commonly found in syntopy with the giant skink. Our analyses suggest that consumption of hard-shelled land crabs is one of the important evolutionary drivers of its large size and high bite force.

## Conclusion

In conclusion, this study highlights the importance of technological advancements (CT and MRI scanning, biomechanics, isotope analyses and genetic prey identification) and integrated studies to unravel long standing evolutionary puzzles of exceptionally rare specimens. Our findings emphasize the role of geographic isolation through insularity of a particularly reduced environment resulting in unique combination of morphological features making *Phoboscincus bocourti* to become an apex predator to successfully exploit all available prey resources.

## Materials and methods

### Ethical statement

Animals in this study were treated in accordance with the laws of France and the French Overseas Territories (Decret 2003-768/NOR: AGRD0300394D). Research protocols, licenses and permission to work and handle (no specimen was collected) were issued and ethically approved by the governments of France and New Caledonia (permits n° 62-2012/ARR/DENV of 9 January 2012 and n° 2806-2012/ARR/DENV of 6 December 2012).

### Field-work and sampling

Two individuals of *Phoboscincus bocourti* (TIS863–TIS864) were hand caught by two of us (MH and II) in December 2012 on the islet where the species lives (New Caledonia) and kept in captivity for 48 h with no food supply. The fresh individual’s fecal samples (one pellet for each) were collected and placed in 95° ethanol for prey identification. After the field work, the samples were placed in petri dishes with alcohol to break free the food items for further visual identification. Several colleagues studying arthropods at Paris Natural History Museum were contacted to identify remains and André Nel (entomologist) identified them as being from a crustacean. Besides the crustacean shell remains no other apparent food items were observed in the feces (Fig. 1).

### Fecal sample DNA identification

Whole-genomic DNA was extracted from the feces using the PureLink Genomic DNA Mini Kit (Invitrogen, Paisley, UK), according to the manufacturer's protocol. COI and 16S rDNA primers were targeted to amplify the crustacean samples. Primers for amplification and PCR conditions are listed in Supplementary Table S1. PCR product purification and sequencing were outsourced to a commercial company (GENEWIZ, Leipzig, Germany).

The obtained sequences were checked and edited using Sequencher v5.4.6 (Gene Codes Corporation, Ann Arbor, MI, USA), and thereafter submitted to GenBank [OM478506 (COI), OM478530 (16S)].

### CT and MRI scanning and segmentation

We used the previously published computerized tomography (CT) data from Caut et al.^12^ to segment the skull and mandible of *Phoboscincus bocourti* holotype (Supplementary Fig. S1) and to take the 3D coordinates of the jaw articulation, muscle insertion sites, and the bite points. In brief, the holotype was scanned on a Viscom X8050-16 μCT scanner at the Center for Microtomography at the University of Poitiers (France) with a voxel size of 74.7 µm. The same specimen was subsequently scanned with magnetic resonance imaging (MRI) with a Bruker Biospec System (Bruker, Germany), at the Institut du Cerveau et de la Moelle épinière, Paris, France, at a voxel size of 100 µm. The MRI scans were then manually segmented using Avizo 8.0 (Visualization Sciences Group, 2013) and muscles were isolated following Wineski and Gans^40^.

### 3D bite model

Our virtual dissection data (X-, Y- and Z-coordinates of origin and insertion and anatomical cross-sectional area) were subsequently used as input for a static bite model. The model used is identical to one previously described^51,54,74–76^ and relies on the computation of the static force equilibrium. The input for the model consists of the 3D coordinates of origin and insertion of the jaw adductors, the physiological cross-sectional area of the jaw muscles, and the 3D coordinates of the point of application of the bite force and the center of rotation. The centroid area of insertion was used for muscle bundles with relatively broad areas of origin and insertion. The coordinates were determined using AVIZO. Complex pennate muscles were separated into their component parts and no correction for pennation was included as the resolution of the MRI scan did not allow us to identify pennation angles. Cross-sectional areas were scaled using a muscle stress estimate of 30 N cm^−1^^77^. Simulations were run at gape angles of 10, 30, and 60 degrees with all jaw adducting muscles set as maximally active for all individuals. Bite forces were calculated at a range of orientations of the food reaction forces, and at four different bite points: the tip of the jaw, the anterior caniniform tooth, the posterior caniniform tooth and the posterior-most tooth. Model output consists of the magnitude of the bite forces and joint forces and the orientation of the joint forces at any given orientation of the food reaction forces.

### In vivo bite forces

To be able to compare the bite force data estimated here for *Phoboscincus bocourti* we assembled a data set for 332 specimens belonging to 44 skink species^52,78–80^. In vivo bite forces were measured in the field or in zoos using an isometric Kistler force transducer (type 9203, range ± 500 N; Kistler, Zurich, Switzerland) mounted on a purpose-built holder and connected to a Kistler charge amplifier (type 5995A, Kistler; see^81^ for a more detailed description of the setup). The place of application of bite forces was standardized for all animals assuring that animals always bit at the same position along the jaw (i.e., at the tip of the jaw). Gape angle was standardized by setting the plates such that all animals bit at a gape angle of about 30°. Measurements were repeated five times for each animal and the maximal value obtained during a recording session was considered to be the maximal bite force for that individual (Fig. 4).

### Prey hardness

We experimentally investigated the force needed to crush food items consumed (the crab *Geograpsus grayi*) by *Phoboscincus bocourti*. The linear dimensions (length and width) and mass of the crab were recorded before crushing. The hardness or resistance was measured using an isometric Kistler force transducer (type 9203, Kistler Inc., Winterthur, Switzerland) connected to a Kistler charge amplifier (type 5995, Kistler Inc.; see^82^). A long screw with a flattened free end (surface area of 3 mm^2^) was mounted on the force transducer, and pushed onto the crab exoskeleton until mechanical failure of its external surface occurred and the maximal force was recorded. We compared data for *Geograpsus grayi* to previously collected data for other crustaceans^83^.

## Supplementary Information

Supplementary Information 1.

Supplementary Information 2.

Supplementary Information 3.
