# Supplementary material for: The Terrific Skink bite force suggests insularity as a likely driver to exceptional resource use
Source: Sci Rep. 2022 Mar 17;12:4596. doi: 10.1038/s41598-022-08148-6 (PMC8930981; doi:10.1038/s41598-022-08148-6)
Supplement: Supplementary file 2 — Supplementary Information 2. [file 41598_2022_8148_MOESM2_ESM.docx]

**Supplementary Table S1**

**Table S1.** Primers and PCR conditions used for amplification of *Geograpsus grayi* individual from fecal samples of *Phoboscincus bocourti*.

| **Primer** | **Sequence (5’-3’)** | **Source** | **PCR conditions** |
| --- | --- | --- | --- |
| **COI** |  |  |  |
| mlCOIintF | GGWACWGGWTGAACWGTWTAYCCYCC | Leray et al. 2013 | 94ºC (4'); [x16] 94ºC (30''),  62ºC (40''), 72ºC (90''); [x25] 94ºC (30''), 46ºC (40''), 72ºC (90''); 72ºC (7') |
| jgHCO2198 | TAIACYTCIGGRTGICCRAARAAYCA | Geller et al. 2013 |  |
| **16S rRNA** |  |  |  |
| Ins16S_1 | TRRGACGAGAAGACCCTATA | Clarke et al. 2014 | 94ºC (4'); [x35] 94ºC (30''), 55ºC (40''), 72ºC (30''); 72ºC (10') |
|  | TCTTAATCCAACATCGAGGTC |  |  |
|  |  |  |  |

**References**

Leray M, Yang JY, Meyer CP, Mills SC, Agudelo N, Ranwez V, Boehm JT, Machida RJ. 2013. A new versatile primer set targeting a short fragment of the mitochondrial COI region for metabarcoding metazoan diversity: application for characterizing coral reef fish gut contents. *Front. Zool.* **10**, 34

Clarke LJ, Soubrier J, Weyrich LS, Alan C. 2014. Environmental L. metabarcodes for insects: in silico PCR reveals potential for taxonomic bias. *Mol. Ecol. Res.* **14**, 1160-1170.

Geller J, Meyer C, Parker M, Hawk H. 2013. Redesign of PCR primers for mitochondrial cytochrome c oxidase subunit I for marine invertebrates and application in all-taxa biotic surveys. *Mol. Ecol. Res*. **13**, 851-861.
