# Supplementary material for: The Terrific Skink bite force suggests insularity as a likely driver to exceptional resource use
Source: Sci Rep. 2022 Mar 17;12:4596. doi: 10.1038/s41598-022-08148-6 (PMC8930981; doi:10.1038/s41598-022-08148-6)
Supplement: Supplementary file 3 [file 41598_2022_8148_MOESM1_ESM.odt]

Supplementary Figures

Figure S1. Phoboscincus bocourti holotype. Photograph by Ivan Ineich.

Figure S2. Isotope values from Phoboscincus bocourti with no correction factor. The letter B is Geograpsus grayi and is shown in the red circle. The red arrow pointed downwards is the corrected isotope discrimination factor published in Caut et al. (2013). 

Figure S3. CT scans of Phoboscincus bocourti holotype head showing dentition.

Figure S4. Image of RX Solution microtomograph of Amphiglossus reticulatus with whole crab claw in stomach. Individual housed in Museo Regionale di Scienze Naturali (Torino) under the catalogue number MRSN R1720 (collected on 15/2/2000 on the Sahamalaza Peninsula, Madagascar).

Figure S5. X-ray Wiscom microtomography showing teeth structure of Phoboscincus bocourti, P. garnieri, and closely related species, Eugongylus albofasciolatus, E. rufescens, Epibator nigrofasciolatus [former Lioscincus nigrofasciolatum]. The top picture of Phoboscincus bocourti is a lateral view of the head with the skin, illustrating morphology, direct rendering volume. All other images are lateral view of the head with a threshold to have an emaciated view, revealing the teeth of the maxillary and premaxillary bones and the jaw.
