# Supplementary material for: The Terrific Skink bite force suggests insularity as a likely driver to exceptional resource use
Source: Sci Rep. 2022 Mar 17;12:4596. doi: 10.1038/s41598-022-08148-6 (PMC8930981; doi:10.1038/s41598-022-08148-6)
Supplement: Supplementary file 4 [file 41598_2022_8148_MOESM3_ESM.odt]

Supplementary Table S2.

Bibliography of lizards predating on crabs.

| Family | Species | Crab (family/genus/species) | Habitat | Reference |
| --- | --- | --- | --- | --- |
| Anguidae | Diploglossus millepunctatus | Grapsus grapsus | Marine | López-Victoria M, Herrón PA, Botello JC. 2011. Notes on the ecology of the lizards from Malpelo Island, Colombia. Bol. Invest. Mar. Cost. 40, 79-89. |
| Anguidae | Ophisaurus ventralis | Uca pugnax | Marine | Watts MW, Steen DA. 2019. Ophisaurus ventralis (Eastern Glass Lizard). Diet. Herpetol. Rev. 50, 788. |
| Dactyloidae | Anolis agassizi | ? | Marine | López-Victoria M, Herrón PA, Botello JC. 2011. Notes on the ecology of the lizards from Malpelo Island, Colombia. Bol. Invest. Mar. Cost. 40, 79-89. |
| Iguanidae | Iguana iguana | Uca spp. | Marine | Mayfield AE, Marcano-Vega H, Lugo AE. 2021. Regional Summaries: Southeast and Caribbean. In: Poland, Therese M.; Patel-Weynand, Toral; Finch, Deborah M.; Ford Miniat, Chelcy; Hayes, Deborah C.; Lopez, Vanessa M., eds. Invasive Species in Forests and Rangelands of the United States: A Comprehensive Science Synthesis for the United States Forest Sector. Heidelberg, Germany: Springer International Publishing: 426-455. Appendix., 426-455. Govender Y, Muñoz MC, Ramirez Camejo LA, Puente-Rolon AR, Cuevas E, Sternber L. 2012. An isotopic study of diet and muscles of the Green Iguana (Iguana iguana) in Puerto Rico. J. Herpet. 46, 167-170. |
| Iguanidae | Cyclura nubila | Cardisoma guandhumi | Land | Beovides-Casas K, Mancina CA. 2006. Natural history and morphometry of the Cuban iguana (Cyclura nubila Gray, 1831) in Cayo Sijú, Cuba. Anim. Biodivers. Conser. 29,  1-8. |
| Iguanidae | Cyclura rileyi | Grapsus grapsus | Marine | Gaskins LC, Silliman BR. 2015. Crab predation by the San Salvador rock iguana (Cyclura rileyi), Herpet. Bull. 132, 27. |
| Iguanidae | Cyclura carinata | Hermit crabs (legs) | Marine | Auffenberg W. 1982. Feeding strategy of the Caicos Ground Iguana, Cyclura carinata. In Iguanas of the World: Their Behavior, Ecology and Conservation. Burghardt, G.M. & Rand, A.S. (Eds.). Noyes Publications, pp 84– 116, Park Ridge, New Jersey, USA. |
| Iguanidae | Ctenosaura similis | Ocypode gaudichaudii | Marine | Arndt RG. 1999. Predation by the black iguana (Ctenosaura similis) on the painted ghost crab (Ocypode gaudichaudii) in Costa Rica. Fla. Scientist 62, 111-114 |
| Leiocephalidae | Leiocephalus carinatus | ? | ? | De Armas L. 1897. Notas sobre la alimentación de Leiocephalus carinatus cayensis (Sauria : Iguanidae). Poeyana 350, 1-7. |
| Scincidae | Amphiglossus astrolabi | ? | Mangrove | Naish D. 2016. The Madagascan skink Amphiglossus eats crabs. Sci. Am. https://blogs.scientificamerican.com/tetrapod-zoology/the-madagascan-skink-amphiglossus-eats-crabs/ |
| Scincidae | Amphiglossus reticulatus | ? | Mangrove | This study |
| Scincidae | Emoia atrocostata | ? ? Small* | Mangrove Marine Land*, mangrove | Huang W-S. 2011. Ecology and reproductive patterns of the littoral skink Emoia atrocostata on an East Asian tropical rainforest island, Zool. Stud. 50, 506-512. Hediger H. 1934. Beitrag zur Herpetologie und Zoogeographie Neu Britanniens und einiger umliegender Gebiete. Zoologische Jahrbücher. Abteilung für Systematik, Ökologie und Geographie der Tiere 65, 441–582. McCoy M. 2006. Reptiles of the Solomon Islands. Pensoft, Sofia-Moscow, Pensoft Series Faunistica n° 57. 147 pp [page 55] |
| Scincidae | Eugongylus albofasciolatus | Small* | Land*, mangrove | McCoy M. 2006. Reptiles of the Solomon Islands. Pensoft, Sofia-Moscow, Pensoft Series Faunistica n° 57. 147 pp [page 70] |
| Scincidae | Eumeces kishinouyei | Picture only available | Freshwater | Unpublished. https://www.mindenpictures.com/search/preview/japanese-skink-eumeces-kishinouyei-eating-crab-iriomote-island-japan/0_00300852.html |
| Scincidae | Eutropis multifasciata | Geosesarma sp | Freshwater | Sy E. 2018. Many‐lined Sun Skink Eutropis multifasciata predation on freshwater crab Geosesarma sp. on Busuanga Island, Philippines. SE Asia Vert Records, 18‐19. |
| Scincidae | Phoboscincus bocourti | Geograpsus grayi | Land | This study |
| Teiidae | Cnemidophorus lemniscatus | ? | ? | Beebe W. 1944. Field notes on the lizards of Kartabo, British Guiana, and Caripito, Venezuela. Part 3. Zoologica 30, 195-216. |
| Teiidae | Kentropyx calcarata | Grapsidae spp. | Mangrove | Roberto IJ, Brito L, Pinto T. 2012. Ecological aspects of Kentropyx calcarata (Squamata: Teiidae) in a mangrove area in northeastern Brazil. Bol. Asoc. Herpetol. Esp. 23, 3-8. |
| Teiidae | Crocodilurus amazonicus | ? | Freshwater | Martins M. 2006. Life in the water: ecology of the jacarerana lizard,Crocodilurus amazonicus. Herpet. J. 16, 171-176 Mesquita DO, Colli GR, Costa GC, França FGR,Garda AA, Peres Jr AK. 2006. At the water´s edge: ecology of semiaquatic Teiids in Brazilian Amazon. J. Herpet. 40, 221-229 |
| Teiidae | Salvator merianae | ? | ? | Abrahão CR, Russell JC, Silva JC, Ferreira F, Dias RA. Population assessment of a novel island invasive: tegu (Salvator merianae) of Fernando de Noronha. Island invasives: scaling up to meet the challenge. 2019;62, 317-25. In: C.R. Veitch, M.N. Clout, A.R. Martin, J.C. Russell and C.J. West (eds.) (2019). Island invasives: scaling up to meet the challenge, pp. 317–325. Occasional Paper SSC no. 62. Gland, Switzerland: IUCN |
| Teiidae | Tupinambis (nigropunctatus) teguixin | ? | ? | Beebe W. 1944. Field notes on the lizards of Kartabo, British Guiana, and Caripito, Venezuela. Part 3. Zoologica 30, 195-216. |
| Teiidae | Pholidoscelis atrata | Coenobita clypeatus | Marine | Donihue CM, Giller GSJ, Herrel A. 2017. Natural history notes: Pholidoscelis atrata (Redonda Ground Lizard). Diet. Herpetol. Rev. 48,  655. |
| Tropiduridae | Microlophus albemarlensis, M. atacamensis | Grapsus grapsus | Marine | Nagu K, Guerra-Correa C, Shoemaker, VH. 2011. Dining Intertidally: Diet, Energetics, and Osmotic Relations of Two Shoreline-Foraging Tropidurid Lizard Species Authors: S. Am. J. Herpetol. 20, 8-16 |
| Varanidae | Varanus albigularis, V. niloticus | Potamonautidae | Freshwater | Dalhuijsen K, Branch WR, Alexander GJ. 2014. A comparative analysis of the diets of Varanus albigularis and Varanus niloticus in South Africa, African Zool. 49, 83-93. |
| Varanidae | Varanus indicus | Cardisoma carnifex, Ocypode spp., Bistro lato | Land, aquatic, coconut | Ehrhard SP. 2016. Dietary behavior of the mangrove monitor lizard (Varanus indicus) on Cocos island, Guam, and strategies for Varanus indicus eradication, Master of Science, University of Hawai at Hilo. |
|  | Kentropyx calcarata | Grapsidae family | Mangrove | Roberto IJ, Brito L, Pinto T. 2012. Ecological aspects of Kentropyx calcarata (Squamata: Teiidae) in a mangrove area in northeastern Brazil, Bol. Asoc. Herpetol. Esp. 23, 3-8. |
| Varanidae | Varanus panoptes, V. mertensi, V. mitchelli, V. gouldii V. mertensi | Holthuisana sp Holthuisana sp | Freshwater | Shine R. 1986. Food habits, habitats and reproductive biology of four sympatric species of varanid lizards in tropical Australia, Herpetologica, 42, 346-360. Mayes PJ, Thompson GG, Withers PC. 2005. Diet and foraging behaviour of the semi-aquatic Varanus mertensi (Reptilia: Varanidae). Wild. Res. 32,  67-74. |
| Varanidae | Varanus salvator | ? Episesarma spp. | ? Marsh | Mahaprom, R. and Kulabtong, S. (2018) Observation of feeding habit of the Asian water monitor, Varanus salvator (Laurenti, 1768) (Squamata Varanidae) on a Asian toad, Duttaphrynus melanostictus (Schneider, 1799) (Anura Bufonidae) in Thailand, Biodivers. J. 9, 213-216. Kulabtong S, Mahaprom R. 2015. Observation on food items of Asian water monitor, Varanus salvator (Laurenti,1768) (SquamataVaranidae), in urban ecosystem, Central Thailand, Biodivers. J. 6, 695-698 |
| Varanidae | Varanus dumerilii | Somanniathelphusa spp and other genera (?) Eriocheir sinensis | Freshwater Freshwater | Technopolis K, Luang K. 2008. Geographical distribution, instinctive feeding behavior and report of nocturnal activity of Varanus dumerilii in Thailand. Biawak 2, 152-158. Krebs U. 1979. Der Dumeril-Waran (Varanus dumerilii), ein spezialisierter Krabbenfresser? (Reptilia: Sauria: Varanidae). Salamandra, 15, 146-157. |
| Varanidae | V. albigularis, V. niloticus | Potamonautidae | Freshwater | Dalhuijsen K, Branch WR, Alexander GJ. 2014. A comparative analysis of the diets of Varanus albigularis and Varanus niloticus in South Africa. Afr. Zool. 49, 83-93. |
| Varanidae | Varanus indicus | Grapsidae | Marine | McCoy M. 2006. Reptiles of the Solomon Islands. Pensoft, Sofia-Moscow, Pensoft Series Faunistica n° 57. 147 pp [page 92] |
| Varanidae | Varanus indicus | ? | ? | Philipp KM, Ziegler T, Böhme W. 2007. Preliminary investigations of the natural diet of six monitor lizard species of the Varanus (Euprepiosaurus) indicus group. In Advances in monitor research III, H.-G. Horn, W. Böhme, and U. Krebs, eds., Rheinbach. Mertensiella, 16, 336-345. |
| Varanidae | Varanus caerulivirens | ? | ? | Philipp KM, Ziegler T, Böhme W. 2007. Preliminary investigations of the natural diet of six monitor lizard species of the Varanus (Euprepiosaurus) indicus group. In Advances in monitor research III, H.-G. Horn, W. Böhme, and U. Krebs, eds., Rheinbach. Mertensiella, 16, 336-345. |
| Varanidae | Varanus cerambonensis | ? | ? | Philipp KM, Ziegler T, Böhme W. 2007. Preliminary investigations of the natural diet of six monitor lizard species of the Varanus (Euprepiosaurus) indicus group. In Advances in monitor research III, H.-G. Horn, W. Böhme, and U. Krebs, eds., Rheinbach. Mertensiella, 16, 336-345. |
| Varanidae | Varanus indicus. V. dumerelii, V. gouldii, V. komodoensis, mertensi, V. mitchelli, V. niloticus, V. panoples, V. rosenbergi, V. salvator, V. semiremex, V. bengalensis | ? | ? | Losos JB, Greene HW. 1988. Ecological and evolutionary implications of diet in monitor lizards. Biol. J. Linn. Soc. 35, 379-407 |
| Varanidae | Varanus grayi | Coenobita rugosa (legs) | Marine | Auffenberg W. 1988. Gray's monitor lizard. (University of Florida Press, Gainesville, Florida, 1988). xi + 419 pp. |
| Varanidae | Varanus mabitang | ? | Land # | Gaulke M, Volker Altenbach A, Demegillo A, Struck U. 2007. On the Diet of Varanus mabitang. Mertensiella 16, 228-239. |
| Varanidae | Varanus salvator | ? | Freshwater | Auffenberg W. 1981. The Behavioral Ecology of the Komodo Monitor. (University Presses of Florida, Gainesville, Florida, USA, 1981). x + 406 pp. |
| Varanidae | Varanus bengalensis | Telphusia sp, Paratelphusa hydrodromus, P. edentate, P. jacquemonti, P. cf. guerini, Barytelphusia curicularis, Potomon simulum, Coenonita rugosa, Ocypode ceratothalamus, S. Ocypoda, Geograpsus grayi, Sesarma foxi, Episesarum versicolor, E. Indicum | Marine, freshwater, land | Auffenberg W. 1994. The Bengal Monitor (University Press of Florida, Gainesville, Florida, 1994). xxviii + 561 pp. |
| Varanidae | Varanus ornatus | Several spp. ? | Mangrove, swamp, forest | Angelici FM, Luiselli L. 1999. Aspects of ecology of Varanus niloticus in south-eastern Nigeria, and their contribution to the knowledge of the evolutionary history of V. niloticus species complex, Rev. Ecol. (La Terre et la Vie) 54, 29-42. |

* small crabs possibly from the genus Parahelice (Family Varunidae) or "mangrove" crabs (Family Sesarmidae (<20 mm) and soft bodied (McCoy personal communication).

 # Unidentified species (local name Kwataw or Man-ok (in Philippine), red land crab (in English).
